# Improving the quality of transurethral resection of bladder tumour (TURBT) operative notes following the European Association of Urology guidelines

**DOI:** 10.1007/s11845-025-03940-5

**Published:** 2025-04-23

**Authors:** Paul C. Ryan, Diarmuid D. Sugrue, Clare O’Connell, Ahmed Salloum, Thomas H. Lynch, Lisa Smyth, Imtiaz Ahmed, John Sullivan, Rustom P. Manecksha, Peter E. Lonergan, Louise C. McLoughlin

**Affiliations:** 1 Department of Urology, St. James’s Hospital, James’s Street, Dublin, Ireland; 2 Department of Surgery, School of Medicine, Trinity College Dublin, Dublin, Ireland

**Keywords:** Guidelines, Operative note, Quality improvement, TURBT, Urothelial cancer

## Abstract

**Background:**

Transurethral resection of bladder tumour (TURBT) is the gold standard for diagnosing and risk-stratifying bladder cancer. Accurate and comprehensive operative documentation is critical for ensuring effective patient management. The European Association of Urology (EAU) guidelines provide a framework for TURBT documentation, including tumour characteristics, procedural details, and complications. However, adherence to these guidelines varies, necessitating quality improvement initiatives.

**Aims:**

This study aimed to assess the quality of TURBT operative notes at a single institution before and after implementing a standardised electronic TURBT template based on EAU guidelines.

**Methods:**

A closed-loop audit was conducted at an academic teaching hospital. Operative notes for 40 consecutive TURBT cases from December 2021 to September 2022 were evaluated against EAU documentation criteria. Following the introduction of a standardised electronic TURBT template, a second audit cycle of 40 cases from September 2022 to March 2023 was conducted. Key documentation elements, including tumour location, appearance, size, multifocality, procedural steps, resection completeness, and complications, were compared.

**Results:**

The introduction of the electronic template significantly improved documentation adherence, increasing overall compliance from 69 to 93%. Notable improvements were observed in tumour appearance (65 to 97.5%), tumour size (67.5 to 90%), completeness of resection (55 to 95%), and complication recording (2.5 to 75%).

**Conclusions:**

A structured electronic TURBT template enhances the quality and completeness of operative documentation, aligning with EAU guidelines. Standardised templates facilitate better communication, continuity of care, and quality improvement in TURBT procedures, ultimately contributing to improved patient management and outcomes.

## Introduction

While the content and format of surgical operative notes has evolved over time, their importance in patient care, disease management, and education remains critical. The Royal College of Surgeons (RCS) maintains that every operative procedure should be documented, including ‘sufficient detail to enable continuity of care’ in their ‘Good Surgical Practice’ guidelines [1].

Transurethral resection of bladder tumour (TURBT) is the mainstay for diagnosing and risk-stratifying bladder cancer by allowing for complete tumour resection, accurate staging, and aids decision-making for individualised treatment plans and long-term patient management [2]. Accurate operative findings and technique documentation are arguably as crucial to patient care as the TURBT.

The European Association of Urology (EAU) has guidelines for TURBT documentation that help characterise, evaluate, and appropriately treat this disease [3]. These guidelines recommend including the following in TURBT operative notes: tumour location, appearance, size and multifocality, all procedure steps, extent, macroscopic completeness of resection, and complications. These variables are also essential in risk-stratifying bladder cancer and guiding adjuvant treatment options. By documenting variables such as tumour size and multifocality, it will help estimate the probability of recurrence of non-muscle invasive bladder cancer (NMIBC) or progression to muscle invasive bladder cancer (MIBC) when used in conjunction with validated risk calculators such as the EAU NMIBC risk calculator [4].

Other studies have explored the implementation of a standardised operative note as a tool for quality improvement in surgical practice. Anderson et al. created a 10-item checklist as a means to improving operative note documentation for the resection of bladder tumours [5]. Guerero et al. performed a similar study investigating the quality of operative notes following EAU guidelines [6].

This study reports the results of a closed-loop audit performed at a single centre on the operative documentation of TURBT outcomes by EAU guidelines, following the introduction of a standardised electronic operative template.

## Methods

This audit was conducted at an academic teaching hospital. The operative notes for 40 consecutive TURBT cases between 1 December 2021 and 30 September 2022 were assessed against the EAU recommendations. Documentation of tumour location, appearance, size, multifocality, all steps of the procedure, extent (interpreted as resection depth), macroscopic completeness of resection, and complication recording were assessed.

TURBT cases for patients > 18 years old undergoing primary TURBT for benign or malignant disease, re-resection, or TURBT for recurrent disease were included. Tumours treated with means other than resection were excluded. Cases were identified from a secure operating theatre database, and procedural notes were accessed from each individual electronic patient record.

**Fig. 1:**
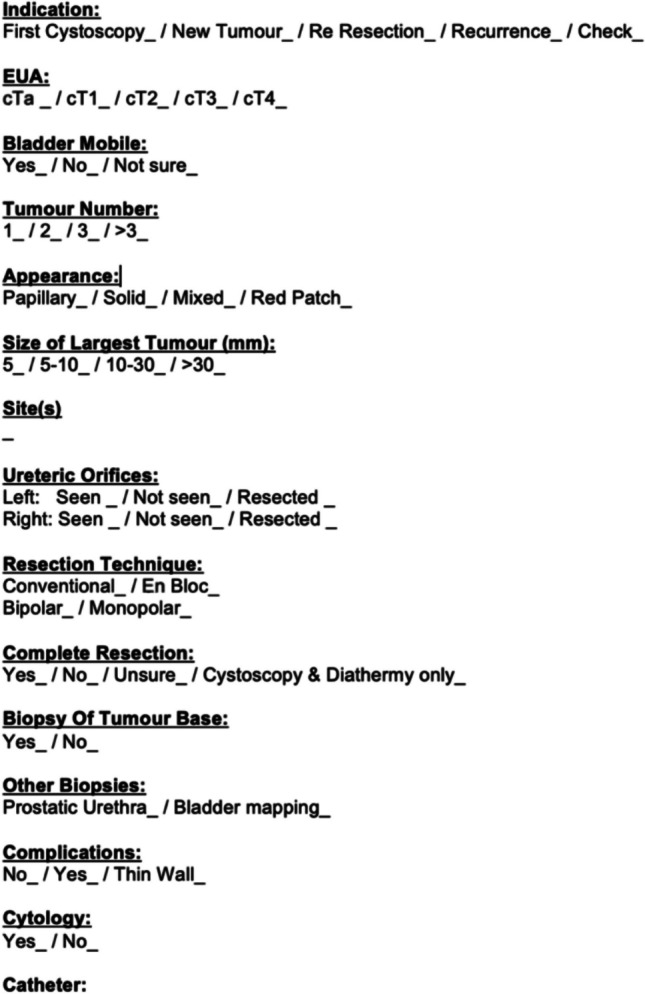
TURBT template

After the initial data analysis, a standardised electronic TURBT template was created in line with EAU guidelines [3] and made available to all operating surgeons (Fig. 1). A further 40 consecutive TURBT procedural notes between September 2022 and March 2023 were re-audited against the same EAU guidelines [3] following the introduction of this template.

This audit was performed following institutional approval (St. James’ Hospital, Dublin 8, Ireland, R&I no. 8306).

## Results

The operative notes for 40 consecutive TURBT procedures involving 36 patients between December 2021 and September 2022 were analysed in the first cycle of the audit. A further 40 operative notes involving 34 patients between September 2022 and March 2023 were analysed in the second audit cycle.

The mean (standard deviation) age of the patients in the first cycle was 68 (10.13) years, compared to 70 (11.95) years for the second cycle. There was a similar sex distribution within both cycles: 75% male in the first cycle and 79% male in the second cycle. Ninety-five percent of operative notes were written by trainees in both cycles. The majority of TURBTs carried out were for primary resections in both cycles (*n* = 19 (47.5%) and *n* = 25 (62.5%) respectively) (Table 1). Malignancy accounted for 87.5% of TURBTs in each cycle (Table 2).

**Table 1:** Patient demographics

	Cycle 1	Cycle 2
Age, median (range)	68 (28–89)	70 (31–93)
Sex: male, *n* (%)	27 (67.5%)	27 (67.5%)
Primary resection, *n* (%)	19 (47.5%)	25 (62.5%)
Re-resection, *n* (%)	4(10%)	6 (15%)
Resection for recurrence, *n* (%)	17 (42.5%)	9 (22.5%)
Consultant note, *n* (%)	2 (5%)	2 (5%)
Trainee note, *n* (%)	38 (95%)	38 (95%)

**Table 2:** Pathological stage

*n* (%)	Cycle 1	Cycle 2
pT0	5	5
pTa low grade	13	17
pTa high grade	3	5
pTis	0	1
pT1 low grade	0	2
pT1 high grade	11	6
pT2	7	4
Other malignancy	1	0

The adherence to EAU guidelines [3] in TURBT documentation improved in cycle 2 following the introduction of the standardised TURBT template. This template was used in 36 of 40 cases in the second cycle. Documentation of tumour location was present in all cases across cycles 1 and 2. Still, improvements were seen in the seven other aspects of the procedural note (Table 3).

**Table 3:** EAU documentation guidelines

*N* (%)	Cycle 1	Cycle 2
Tumour location	100% (*n* = 40)	100% (*n* = 40)
Tumour appearance	65% (26)	97.5% (39)
Tumour size	67.5% (27)	90% (36)
Tumour multifocality	92.5% (37)	97.5% (39)
All steps of the procedure	85% (34)	92.5% (37)
Extent (interpreted as resection depth)	85% (34)	97.5% (39)
Resection completeness	55% (22)	95% (38)
Complications	2.5% (1)	75% (30)

There was an increase in tumour appearance documentation from 65 to 97.5% between cycles 1 and 2, tumour size documentation from 67.5 to 90%, and tumour multifocality documentation from 92.5 to 97.5% across both cycles. All procedure steps were documented in 85% of the cases in the first cycle compared to 92.5% in the second. The extent of resection documentation rose from 85 to 97.5% across both cycles, and completeness was documented in 55% of cases in cycle 1 and 95% of cases in cycle 2. Documentation of complications was present in 2.5% of cases in cycle 1 and 75% in cycle 2.

On a global assessment of operative documentation between both cycles, the average documentation rate for cycle 1 was 69% and 93% for cycle 2.

## Discussion

The aim of this study was to evaluate the impact of a standardised electronic operative note template on the quality of TURBT documentation in accordance with European Association of Urology (EAU) guidelines [3]. Through a closed-loop audit conducted at a single academic centre, we compared TURBT operative notes before and after the implementation of this template. The findings demonstrated a significant improvement in documentation adherence, with overall compliance increasing from 69% in the first cycle to 93% in the second. Notable enhancements were observed in key aspects such as tumour appearance (from 65 to 97.5%), tumour size (from 67.5 to 90%), completeness of resection (from 55 to 95%), and complication recording (from 2.5 to 75%). These results highlight the effectiveness of structured operative note templates in improving surgical documentation, ensuring more comprehensive clinical records, and potentially enhancing patient care and treatment planning.

The improvement in documentation observed in the second cycle can be attributed to the creation of the standardised electronic template that was made readily available to all surgeons at the centre. The template was specifically designed in line with the EAU guidelines for documentation and helped to ensure that all key procedural details were consistently reported. In four (10%) of the cases in the second cycle, the template was not used for reasons not known to the author.

A TURBT has the potential to be diagnostic, prognostic, and curative. The value of a TURBT has been understood for decades and due to the link between TURBT and bladder cancer prognosis, several quality improvement studies have been reported in the literature [5–8]. Guerero et al. performed a similar study investigating the quality of operative notes following EAU guidelines and reported improvements in all but one aspect of documentation between cycles 1 and 2 [6]. This current study differs from that by Guerero by focusing on 80 consecutive TURBT cycles between both cycles with no break in between. Unlike Guerero et al., this study did not report specifically on the instillation of post-operative intravesical treatment nor ‘bimanual examination under anaesthesia’. The latter is, however, included under the documentation of ‘all steps of the procedure’. Both of these studies validate the advantage of an operative template in ensuring adequate documentation in TURBT procedures.

Recently, the RESECT (transurethral resection and single instillation intra-vesical chemotherapy evaluation in bladder cancer treatment) study, a global multi-centre observational audit of urological practice relating to non-muscle invasive bladder cancer (NMIBC), investigated whether audit and feedback can help improve the quality of treatment of NMIBC by measuring key quality indicators in transurethral bladder tumour resection [9]. These quality indicators include detrusor muscles in the specimen, documentation of tumour number, size and location, the documentation of completeness of resection, and the use of a single instillation of intravesical chemotherapy within 24 h of the TURBT.

Medical record-keeping has many ramifications for the patient and the healthcare practitioner. Clear documentation allows for sufficient continuity of care, communication between medical stakeholders and, most importantly, accurate diagnosis and prognosis of disease processes. Operative documentation varies on both an individual and institutional basis; thus, there have been efforts to create operative templates across multiple specialities [10–12]. In a large study by Buchanan et al., the quality of operative reporting of colorectal cancer-specific measures was compared between a pre-determined, fixed template operative note and those documented in narrative operative reports [13]. They found that the template reports were superior to the narrative reports with 84% of all quality measures included in the template reports compared to 43% in the narrative reports. Similarly, this study found that the quality measures improved in all but one domain, with ‘tumour location’ documented in 100% of operative notes in both cycles.

The advantage of electronic templates is not just limited to the template itself. While this may help prevent the operative surgeon from neglecting to record an aspect of the TURBT, having these templates electronically stored also ensures their constant availability, something that may not be guaranteed in every operating theatre with hardcopy templates. Studies have shown the advantage of electronic records in health care [14, 15], but electronic note-keeping is not available in every operating theatre due to a lack of infrastructure. Electronic records allow for enhanced access and availability, ease of information retrieval, streamlined workflow, and a reduced administrative burden. It was found that all operating surgeons easily accessed the surgical template incorporated in the electronic patient record system. The template ensured that the recommendations made by the EAU were presented to the reporting surgeon once it was accessed. This allowed for documentation of all key aspects of the procedure provided that the surgeon responded to the prompts. We found that documentation was near 100% in all areas in cycle two with only 75% achieved for documentation of ‘complications’. Following a departmental progress review, it became clear that there was uncertainty as to whether the surgeon was required to respond to this prompt if there were no complications encountered; thus, this prompt was further developed to include the options of ‘yes/no/thin wall/other’.

There are certain limitations to this study. The study had a relatively small sample size of 80 cases, which would limit generalisability and statistical power in detecting differences or broader applicability.

It was found that although there is an option to provide a pictorial description electronically, it is often not to the satisfaction of the operating surgeon. Further development in this particular area is required. One surgeon also found the template cumbersome and not as user-friendly, given the many prompts presented once the electronic template was accessed. There is a possibility of bias with feedback received from the aforementioned RESECT study [9]. Surgeons may have been influenced by feedback from the RESECT study, leading to increased conscientious efforts to improve documentation, which could inflate the impact of the template. The study did not assess oncological outcomes, so it is unclear whether better documentation led to improved patient care or clinical decision-making. Furthermore, the impact on intravesical recurrence was not assessed.

## Conclusion

Standardised templates help ensure more accurate operative procedure documentation in line with international guidelines. Electronic records facilitate ease of access, storage, and replication of these guidelines. Introducing a standardised, electronic TURBT template is easy to produce and replicate. It leads to improved documentation of the critical aspects of this important procedure, thus aiding the risk stratification of bladder cancer.

## Data Availability

All data is available upon request.
